# Visible-light-driven CO_2_ reduction on a hybrid photocatalyst consisting of a Ru(ii) binuclear complex and a Ag-loaded TaON in aqueous solutions†

**DOI:** 10.1039/c6sc00586a

**Published:** 2016-03-23

**Authors:** Akinobu Nakada, Takuya Nakashima, Keita Sekizawa, Kazuhiko Maeda, Osamu Ishitani

**Affiliations:** a Department of Chemistry, Graduate School of Science and Engineering, Tokyo Institute of Technology 2-12-1-NE-1 O-okayama, Meguro-ku Tokyo 152-8550 Japan ishitani@chem.titech.ac.jp

## Abstract

A hybrid photocatalyst consisting of a Ru(ii) binuclear complex and a Ag-loaded TaON reduced CO_2_ by visible light even in aqueous solution. The distribution of the reduction products was strongly affected by the pH of the reaction solution. HCOOH was selectively produced in neutral conditions, whereas the formation of HCOOH competed with H_2_ evolution in acidic conditions. Detailed mechanistic studies revealed that the photocatalytic CO_2_ reduction proceeded *via* ‘Z-schematic’ electron transfer with step-by-step photoexcitation of TaON and the photosensitizer unit in the Ru(ii) binuclear complex. The maximum turnover number for HCOOH formation was 750 based on the Ru(ii) binuclear complex under visible-light irradiation, and the optimum external quantum efficiency of the HCOOH formation was 0.48% using 400 nm monochromic light with ethylenediaminetetraacetic acid disodium salt as a sacrificial reductant. Even in aqueous solution, the hybrid could also convert visible-light energy into chemical energy (Δ*G*^0^ = +83 kJ mol^−1^) by the reduction of CO_2_ to HCOOH with methanol oxidation.

## Abstract 2

A hybrid photocatalytic system consisting of a Ru(ii) binuclear complex and Ag-loaded TaON can reduce CO_2_ to HCOOH by visible light irradiation even in aqueous solution (TON_HCOOH_ = 750, Φ_HCOOH_ = 0.48%).

## Graphical abstract

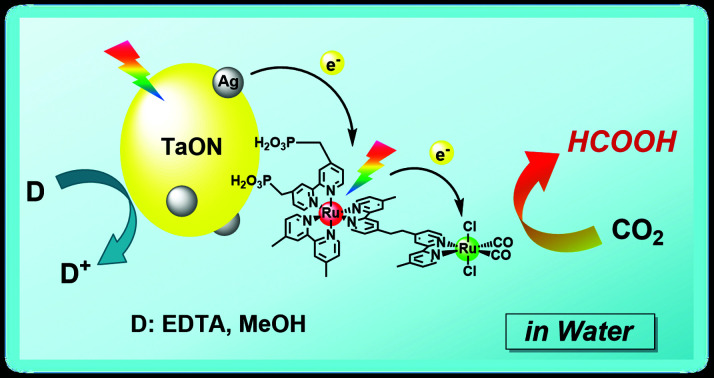

## Introduction

The development of photocatalytic systems for CO_2_ reduction is an attractive research target in the field of conversion of solar energy into chemical energy, the so-called artificial photosynthesis. Artificial photosynthetic reactions have various potential functions; one of these is to use water as both an electron source and as a solvent because water is an abundant and low-cost material. Since both CO_2_ and water are very stable compounds, these photocatalytic systems should have both strong reduction and oxidation power. Utilization of visible light is another important function for artificial photosynthesis because it covers *ca.* 50% of the solar energy, whereas the light in the UV region (*λ* < 400 nm) is very minor (<6%). However, there are few visible-light-driven photocatalysts for CO_2_ reduction which function well in water.

Multinuclear Ru(ii) and/or Re(i) diimine (N^N) complexes with a redox photosensitizer (PS) and a catalyst (CAT) unit, the so-called supramolecular photocatalysts, have attractive abilities as photocatalysts for CO_2_ reduction because of their high efficiencies and selectivities for reducing CO_2_ to HCOOH and CO not only in organic solution^1–9^ but also in aqueous solution.^10,11^ Since proton reduction to H_2_ is a more thermodynamically favorable reaction than CO_2_ reduction, this specific selectivity is a superior property for constructing an artificial photosynthesis system with CO_2_ reduction in aqueous solution. However, the photocatalytic systems constructed with only metal complexes generally require a strong reductant such as NADH model compounds^2,5–9^ and benzimidazoline derivatives^1,3,4,11^ because the excited metal complexes have relatively weak oxidizing power. To add the stronger photooxidizing power, the metal complex photocatalyst should be combined with another photocatalyst for the oxidation reaction.

Some powder semiconductor photocatalysts with much stronger oxidizing power have been reported, which can oxidize even water involving reduction of electron acceptors.^12^ Metal oxynitrides are typical examples; they have sufficient positive valence band potential to oxidize weak reductants and relatively small band gaps to utilize visible light.^13^

**Scheme 1:**
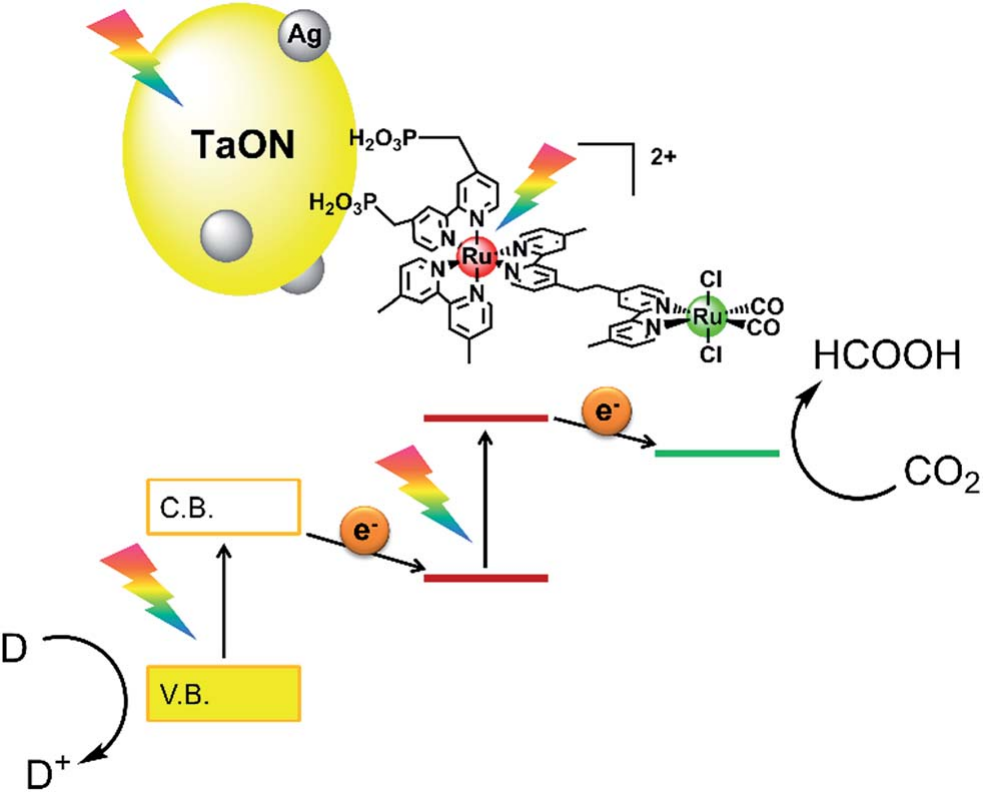
Hybrid powder photocatalyst of the Ru(ii) binuclear complex adsorbed on Ag-modified TaON (RuRu/Ag/TaON).

Based on these investigations regarding the strong and weak points of different types of photocatalysts, we have developed novel hybrid photocatalytic systems where supramolecular photocatalysts connect with metal oxynitride photocatalysts to utilize both the outstanding features of high selectivity and efficiency for CO_2_ photoreduction (supramolecular site) and strong photooxidizing power (semiconductor site). Visible-light irradiation to the hybrid photocatalysts consisting of a Ru(ii) binuclear complex (RuRu) with [Ru(N^N)_3_]^2+^ as the PS unit and Ru(N^N)(CO)_2_Cl_2_ as the CAT unit, which was adsorbed on a tantalum(v) oxynitride (TaON) photocatalyst in pure methanol without any other reductant under a CO_2_ atmosphere, caused the catalytic formation of HCOOH as a reduced product of CO_2_ and formaldehyde as an oxidized product of methanol (MeOH).^14^ Using CaTaO_2_N instead of TaON in the hybrid achieved high selectivity of HCOOH formation (>99%) in dimethylacetamide–triethanolamine mixed solution; meanwhile, the photocatalytic reaction requires a sacrificial electron donor.^15^ These reactions are driven *via* the two-step photoinduced electron transfer mechanism, the so-called ‘Z-scheme’, as shown in Scheme 1: (1) step-by-step photoexcitation of the semiconductor and the Ru(ii) PS unit occurs; (2) the valence band holes are consumed by a reductant; (3) conduction band electrons in the semiconductor transfer to the excited state of the PS unit, producing one-electron-reduced species (OERS) of PS; (4) intramolecular electron transfer from the OERS of the PS unit to the ground state of the CAT unit occurs, producing the reduced CAT unit and (5) CO_2_ reduction proceeds on the reduced CAT.

Along with the Z-scheme hybrid photocatalysts, another powder hybrid photocatalyst consisting of a mononuclear metal complex as the CAT and a semiconductor such as carbon nitride^16–18^ or nitrogen-doped Ta_2_O_5_ ^19,20^ working as a PS have been developed for use in CO_2_ reduction.

However, these hybrid photocatalysts were investigated only in organic solutions; we do not have any information on their photocatalytic activity in water. In this work, the photocatalytic activity of the hybrid photocatalyst of Ag-modified TaON and the Ru(ii) binuclear complex (RuRu/Ag/TaON, Scheme 1) was investigated for the first time in aqueous solutions containing electron donors, and we observed that RuRu/Ag/TaON photocatalyzed efficient CO_2_ reduction with high durability. This Z-schematic hybrid photocatalyst could also drive an uphill reaction, *i.e.* CO_2_ reduction with methanol as a reductant, in a water–methanol mixed solution.

## Results and discussion

**Fig. 1:**
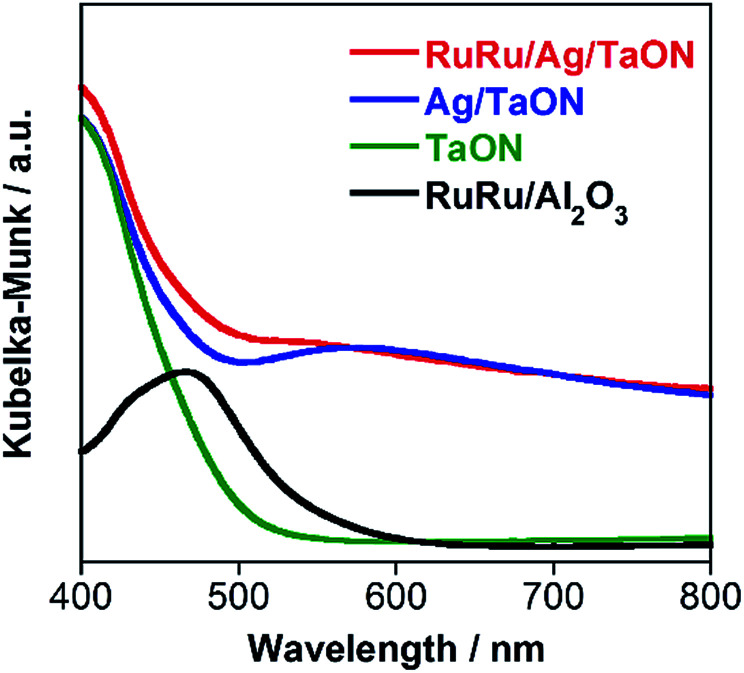
DRS of RuRu/Ag/TaON (red), Ag/TaON (blue), TaON (green) and RuRu/Al_2_O_3_ (black).

A hybrid photocatalyst of Ag-modified TaON and a Ru(ii) binuclear complex RuRu/Ag/TaON was synthesized according to a reported method.^14^ Typically, the loaded amount of silver and RuRu were 1.5 wt% and 3 μmol g^−1^, respectively, except for the experiment corresponding to Fig. 5. The obtained materials were characterized by diffuse reflectance spectroscopy (DRS), X-ray diffraction (XRD), emission spectroscopy and Fourier-transform infrared (FT-IR) spectroscopy, as shown in Fig. 1 and S1–S3, ESI.† The XRD patterns of TaON, Ag/TaON and RuRu/Ag/TaON confirm that the crystal structure of TaON was not changed during the attachment procedures of silver and RuRu on TaON (Fig. S1a, ESI†). The typical diffraction peak at 2*θ* = 38.1° is attributed to metallic silver; this peak appears in the spectra of Ag/TaON and RuRu/Ag/TaON (Fig. S1b, ESI†). Fig. 1 shows DRS spectra of the hybrids RuRu/Ag/TaON, Ag/TaON and TaON along with RuRu/Al_2_O_3_, which is a model of RuRu. A broad absorption band was observed in the cases of Ag/TaON and RuRu/Ag/TaON, which is due to surface plasmon resonance of the metallic silver particles on the surface of TaON. RuRu/Ag/TaON also exhibited an absorption attributable to the Ru(ii) photosensitizer unit (Fig. 1 and S4, ESI†). A dispersion of RuRu/Ag/TaON in water showed emission with *λ*_em_ = 629 nm by photoexcitation at *λ*_ex_ = 444 nm, which is attributable to phosphorescence from the triplet metal-to-ligand charge transfer (^3^MLCT) excited state of the Ru(ii) PS unit as well as phosphorescence from RuRu dissolved in water (Fig. S2, ESI†). IR absorption bands corresponding to the CO stretching vibrations of the Ru(ii) CAT unit were observed at 2061 and 1997 cm^−1^ in the FT-IR spectrum of RuRu/Ag/TaON (Fig. S3, ESI†). These spectroscopic results indicate that the structure of RuRu was maintained after adsorption on Ag/TaON.

**Fig. 2:**
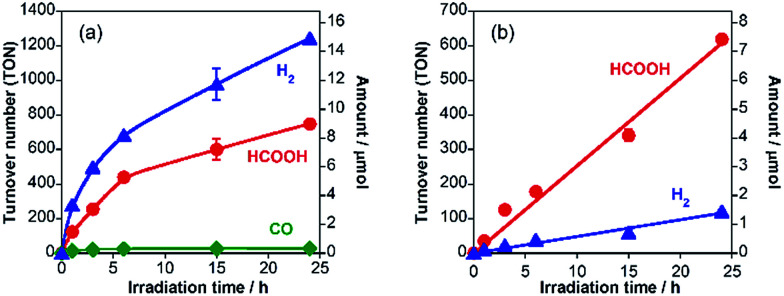
Time courses of HCOOH (red), H_2_ (blue) and CO (green) formation by visible-light (*λ* > 400 nm) irradiation to RuRu/Ag/TaON (4 mg) in EDTA·2Na (10 mM) aqueous solution (4 mL) without (a) and with (b) Na_2_CO_3_ (0.1 M) under a CO_2_ atmosphere.

As a typical run, a powder of RuRu/Ag/TaON (4 mg) was dispersed in aqueous solution (4 mL) containing ethylenediaminetetraacetic acid disodium salt (EDTA·2Na, 10 mM) and irradiated at *λ*_ex_ > 400 nm under a CO_2_ atmosphere. After 24 h irradiation, formic acid, H_2_ and a small amount of CO were produced with turnover numbers (TON) of 750 (8.5 μmol), 1240 (14.2 μmol) and 30 (0.3 μmol), respectively (Fig. 2a). The external quantum yields (*Φ*) of the photocatalytic reaction were *Φ*_HCOOH_ = 0.47% and *Φ*_H_2__ = 0.54% using 400 nm monochromatic light. In contrast, formic acid was produced with much higher selectivity (85%) by addition of Na_2_CO_3_ (0.1 M) to the reaction solution (Fig. 2b), although TON_HCOOH_ (620) and *Φ*_HCOOH_ (0.23%) were lower than those in the absence of Na_2_CO_3_. Details of this difference are described in a later part of this paper.

**Fig. 3:**
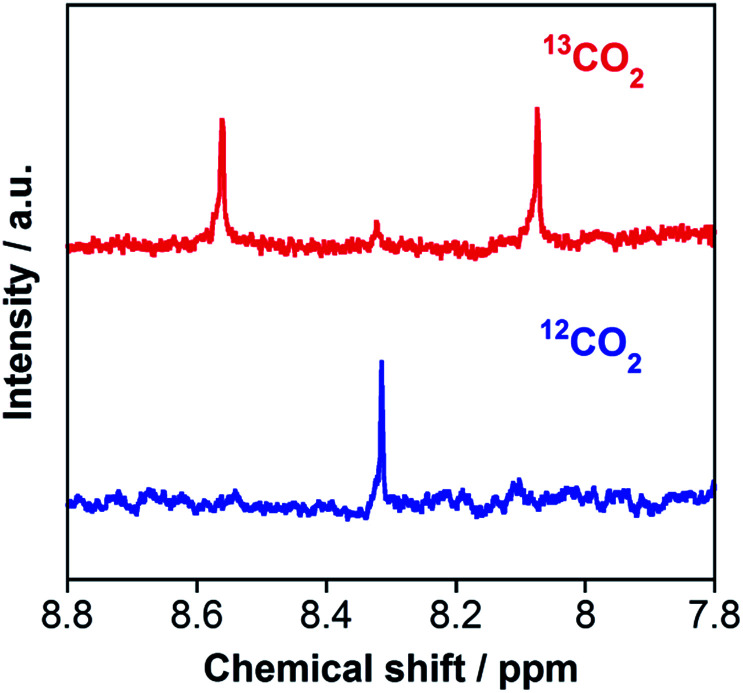
^1^H NMR spectra of the aqueous reaction solutions (1 mL) containing RuRu/Ag/TaON (4 mg) and EDTA·2Na (10 mM), measured after 24 h irradiation at *λ*_ex_ > 400 nm under ^13^CO_2_ (red) and ^12^CO_2_ (blue) atmospheres.

The carbon source of HCOOH was confirmed by an isotope-labeling experiment. A red line in Fig. 3 shows the ^1^H NMR spectrum of the reaction solution after the photocatalytic reaction under the same condition as that described above, except using ^13^CO_2_ instead of ordinary CO_2_. A doublet attributed to H^13^COOH was mainly observed at 8.31 ppm (^1^*J*_CH_ = 196 Hz), with a small singlet attributed to H^12^COOH. In contrast, only a singlet of H^12^COOH was observed for the photocatalysis under ordinary CO_2_ atmosphere (a blue line in Fig. 3). Based on the areas of these peaks, we calculated that 97% of HCOOH was formed by reduction of CO_2_ in the photocatalytic reaction. Notably, this value is comparable with the purity of the ^13^CO_2_ used (99%).

Table 1 summarizes the results of the photocatalytic reactions using various hybrids in aqueous solution containing EDTA·2Na (10 mM). Irradiation to RuRu/Ag/Al_2_O_3_, where Al_2_O_3_ was used as an insulator instead of TaON, did not yield any reduction products (entry 2, Table 1). The oxidizing power of the excited photosensitizer unit in RuRu was evaluated by emission measurements using EDTA·2Na as a quencher (Fig. S5, ESI†). Only 7% of the emission from the ^3^MLCT excited state of the PS unit of RuRu on the surface of Al_2_O_3_ was quenched by 10 mM of EDTA·2Na. These results suggest that EDTA·2Na mainly supplies electrons to the Ag/TaON unit in the photocatalytic reaction using RuRu/Ag/TaON. After the photocatalytic reaction using RuRu/Ag/TaON, we could not observe N_2_ generation by gas chromatography. Furthermore, there were no differences in either the binding energy for the Ta4p peak or the ratio of areas for Ta4p and N1s of TaON in RuRu/Ag/TaON before and after the photocatalytic reaction by X-ray photoelectron spectroscopy (XPS) analysis (Fig. S6, ESI†). These observations indicate that the TaON unit in RuRu/Ag/TaON did not decompose during the photocatalytic reaction, which occasionally becomes a problem in some photocatalytic systems because it consumes photo-generated holes by the decomposition of TaON itself (eqn (1)).^21–25^

1 2N^3−^ + 6h^+^ → N_2_

**Table 1:** Photocatalytic reactions using various hybrids under a CO_2_ atmosphere^a^

Entry	Photocatalyst	Product/μmol (TON)		
		HCOOH	CO	H_2_
1	RuRu/Ag/TaON	7.0 (600)	0.3 (28)	11.4 (978)
2	RuRu/Ag/Al_2_O_3_	N.D.	N.D.	N.D.
3	Ag/TaON	N.D.	N.D.	0.4 (—)
4	RuRu/TaON	1.2 (103)	0.2 (16)	5.0 (420)
5	Ru(Cat)^b^/Ag/TaON	<0.1 (—)	N.D.	<0.1 (—)
6	Ru(PS)^c^/Ag/TaON	<0.1 (—)	N.D.	4.2 (371)

a Dispersion of a photocatalyst (4 mg) in an EDTA·2Na (10 mM) aqueous solution (4 mL) was irradiated at *λ*_ex_ > 400 nm for 15 h.

b Ru(Cat) = *cis*-Ru{4,4′-(CH_2_PO_3_H_2_)_2_-2,2′-bipyridine}(CO)_2_Cl_2_.

c Ru(PS) = [Ru(dmb)_2_{4,4′-(CH_2_PO_3_H_2_)_2_-2,2′-bipyridine}](PF_6_)_2_.

Silver particles have been reported to act as a co-catalyst for CO_2_ reduction on some semiconductor photocatalysts which require irradiation of UV light.^26–34^ However, Ag/TaON without RuRu did not photocatalyze CO_2_ reduction at all (entry 3 in Table 1), indicating that the silver particles of RuRu/Ag/TaON did not work as a co-catalyst for CO_2_ reduction. However, loading silver to the surface of TaON dramatically enhanced the photocatalytic activity of RuRu/Ag/TaON, particularly for CO_2_ reduction (compare entries 1 and 4, Table 1). It has been reported that loading of Ag on the surface of a hybrid photocatalyst RuRu/CaTaO_2_N enhances the photoinduced electron transfer from the conduction band of CaTaO_2_N to the excited states of the Ru photosensitizer unit.^15^ A similar phenomenon should accelerate the photocatalytic ability of RuRu/Ag/TaON in the present system.

Use of the mononuclear model complex of the CAT unit (Ru(Cat)) instead of RuRu drastically lowered the photocatalytic activity of the hybrid (entry 5, Table 1). This is reasonable because the electron transfer from the conduction band of TaON (*E*_CBM_ = −1.31 V)^14^ to Ru(Cat) (*E*^red^_p_ = −1.46 V *vs.* Ag/AgCl at pH 7)^14^ is an endergonic reaction. A hybrid without the catalyst unit (Ru(PS)/Ag/TaON), *i.e.* a mononuclear model complex of the PS unit (Ru(PS)) adsorbed on Ag/TaON, produced a catalytic amount of H_2_ with a very small amount of HCOOH (entry 6, Table 1). There have been some reports that [Ru(N^N)_3_]^2+^-type complexes decompose *via* photoinduced-ligand-substitution reactions to produce [Ru(N^N)_2_(X)(Y)]]^*n*+^-type complexes,^35,36^ and the product [Ru(N^N)_2_(X)(Y)]]^*n*+^ acts as a catalyst for both H_2_ evolution and CO_2_ reduction with the residual [Ru(N^N)_3_]^2+^ as the photosensitizer.^10,11,37^ From these control experiments and the emission quenching measurements, we can conclude that all of the units in the hybrid photocatalyst RuRu/Ag/TaON are necessary for the efficient photocatalytic reduction of CO_2_. RuRu/Ag/TaON worked *via* the Z-schematic electron-transfer mechanism from EDTA·2Na to the Ru catalyst unit with visible-light photoexcitation of both TaON and the Ru photosensitizer unit with the assistance of the Ag particles on the surface of TaON, followed by the CO_2_ reduction proceeding on the Ru catalyst unit, as shown in Scheme 1.

**Fig. 4:**
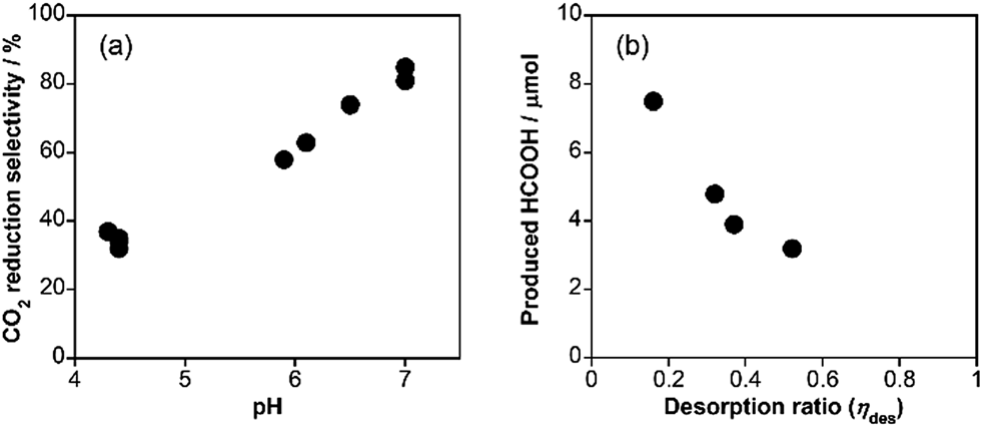
(a) Selectivity of CO_2_ reduction (sel_CO_2__) *vs.* pH of the reaction solution in the photocatalytic reactions. (b) Produced amount of HCOOH *vs.* desorption ratio of RuRu (*η*_des_) by the photocatalytic reactions with various concentration of NaH_2_PO_4_ (pH = 4.4).

The effects of coexistent ions and the pH of the reaction solution on the photocatalytic activity were examined in detail with a series of additional salts to the reaction solution. Table 2 summarizes the results using EDTA·2Na (10 mM) as an electron donor, including the produced amounts of the reduction products, the selectivity of CO_2_ reduction (sel_CO_2__) and the desorption ratios of the surface-bound RuRu (*η*_des_). Addition of Na_2_CO_3_ (entry 2 in Table 2), K_2_CO_3_ (entry 3) and Na_2_HPO_4_ (entry 4), which changed the pH of the reaction solution to between 6.5 and 7.0, dramatically improved the selectivity of CO_2_ reduction. On the other hand, the change in ion strength of the reaction solution should not be a reason for this change in selectivity because the selectivity did not change in reaction solutions containing various concentrations of NaH_2_PO_4_ (34–35%, pH = 4.4, entries 5–7), where the pH was similar to that without the salts (37%, pH = 4.3, entry 1). Fig. 4a (plots of entries 1–8 and 11) exhibit clear trend that higher pH increased the selectivity of CO_2_ reduction unrelated to the ion strength of the solution; a more basic solution suppresses the evolution of H_2_, probably because of the lower proton concentration in the reaction solution.

**Table 2:** Results of photocatalytic reactions using RuRu/Ag/TaON (4 mg) in EDTA·2Na (10 mM) aqueous solutions containing various salts (4 mL) under visible-light (*λ* > 400 nm) irradiation for 15 h

Entry	Salt^a^	pH^b^	Product/μmol (TON)			sel_CO_2__^c^/%	*η*_des_/%
			HCOOH	CO	H_2_		
1	None	4.3	7.0 (600)	0.3 (28)	11.4 (978)	37	17
2	Na_2_CO_3_	7.0	4.0 (340)	N.D.	0.7 (60)	85	58
3	K_2_CO_3_	7.0	3.7 (307)	N.D.	0.9 (74)	81	60
4	Na_2_HPO_4_	6.5	5.6 (482)	<0.1	2.0 (172)	74	58
5	NaH_2_PO_4_	4.4	3.2 (257)	<0.1	6.1 (481)	35	52
6	NaH_2_PO_4_^d^	4.4	3.9 (327)	<0.1	7.9 (658)	34	37
7	NaH_2_PO_4_^e^	4.4	4.8 (421)	0.2 (13)	9.0 (791)	34	32
8	Na_2_HPO_4_^f^ + NaH_2_PO_4_^f^	6.1	4.8 (418)	<0.1	2.8 (245)	63	53
9^g^	None	4.3	0.7 (56)	N.D.	0.5 (46)	32	—
10^h^	None	4.3	1.3 (350)	<0.1	2.9 (773)	55	—
11^i^	None	5.9	6.7 (589)	0.1 (12)	4.8 (418)	58	26

a Concentration was 0.1 M except for entries 6–8.

b After purging with CO_2_ for 20 min.

c Selectivity of CO_2_ reduction.

d Concentration was 0.03 M.

e Concentration was 0.01 M.

f Concentration was 0.05 M.

g Using Ag/TaON (4 mg) and Ru(bpy)_2_(CH_3_bpyCH_2_CH_2_bpyCH_3_)Ru(CO)_2_Cl_2_ (12 nmol).

h Adsorption amount of RuRu was 1.0 μmol g^−1^.

i Using ethylenediaminetetraacetic acid tetrasodium salt (EDTA·4Na, 10 mM) instead of EDTA·2Na.

The produced amounts of HCOOH were lowered by the addition of the salts (0.1 M), regardless of the solution pH (entries 2–5). The UV-vis absorption spectra of the filtrates of the reaction solutions after the photocatalytic reactions exhibit an absorption band attributed to RuRu (Fig. S7, ESI†), indicating that RuRu partially desorbed from RuRu/Ag/Al_2_O_3_ during the photocatalytic reaction. Ru(ii) diimine complexes with phosphonic acid anchor groups have been widely used as a photosensitizer in various photocatalytic systems^14–19,38–41^ and dye-sensitized photoelectrochemical cells.^42–50^ It was reported that in many cases, desorption of Ru complexes from the surface of metal oxides proceeded under visible-light irradiation in aqueous solution.^51–54^ The *η*_des_s were 52–60% in the presence of the salts (0.1 M; entries 2–5), which were three times larger than those in the absence of the salts (entry 1). Higher concentration of salts in the reaction solution induced higher *η*_des_ and lower TON (Fig. 4b), while lower concentration of salts suppressed the desorption of the metal complex and deactivation of the photocatalytic reaction (entries 1 and 6–8 in Table 2 and Fig. 4b). On the other hand, the pH of the solution and the type of added salts did not strongly affect *η*_des_ (entries 2–5). A mixed system of Ag/TaON (4 mg) and a Ru(ii) binuclear complex without the methyl phosphonate anchoring groups (12 nmol) showed much lower photocatalytic abilities (compare entry 1 and entry 9). Therefore, the addition of salts accelerated the desorption of RuRu, lowering the photocatalytic activity of RuRu/Ag/TaON. This is also supported by the following experimental data: the use of RuRu/Ag/TaON with a smaller amount of RuRu (1.0 μmol g^−1^) produced much smaller amounts of HCOOH and H_2_ (1.3 and 2.9 μmol, entry 10) compared with RuRu/Ag/TaON with 3.0 μmol g^−1^ of RuRu (7.0 μmol of HCOOH and 11.4 μmol of H_2_, entry 1). The details of the effects of the adsorbed amount of RuRu on the activity are described later. Taking into account these effects of pH and concentration of additives, higher selective HCOOH formation (58% selectivity) was obtained when ethylenediaminetetraacetic acid tetrasodium salt (EDTA·4Na, pH = 5.9; entry 11) was used instead of EDTA·2Na (pH = 4.3; entry 1) keeping high TON of 589 for HCOOH formation.

**Fig. 5:**
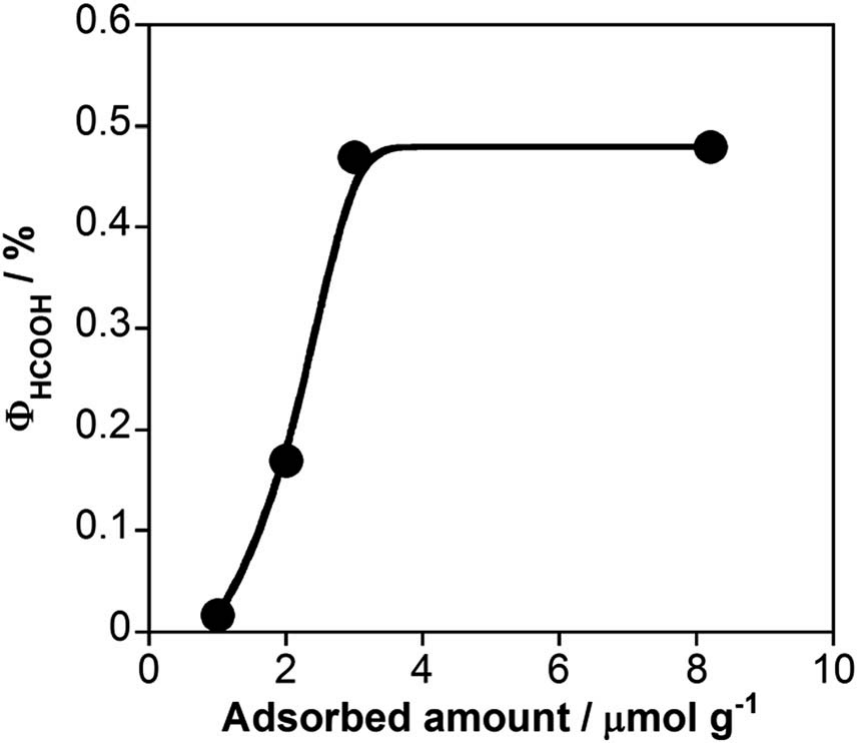
Relationship between *Φ*_HCOOH_ and loading amount of RuRu in the photocatalytic reaction using RuRu/Ag/TaON (30 mg) and EDTA·2Na (10 mM) in aqueous solution (10 mL) with 400 nm monochromatic light irradiation under a CO_2_ atmosphere.

Fig. 5 shows the external quantum efficiencies for photocatalytic HCOOH production (*Φ*_HCOOH_) using RuRu/Ag/TaON with various loading amounts of RuRu. The *Φ*_HCOOH_ increased with increasing loading amount of RuRu from 1.0 to 3.0 μmol g^−1^ and then reached plateau with the maximum values of *Φ*_HCOOH_ = 0.48% at 8.3 μmol g^−1^. This is probably why the separation of the electron–hole pairs in TaON was accelerated because of the electron transfer from the conduction band to RuRu. The loading amount of 3.0 μmol g^−1^ might be sufficient to produce this effect. Notably, *Φ*_HCOOH_ is the highest value obtained for photocatalytic CO_2_ reduction using semiconductor–photosensitizer–catalyst triad systems to date.

**Fig. 6:**
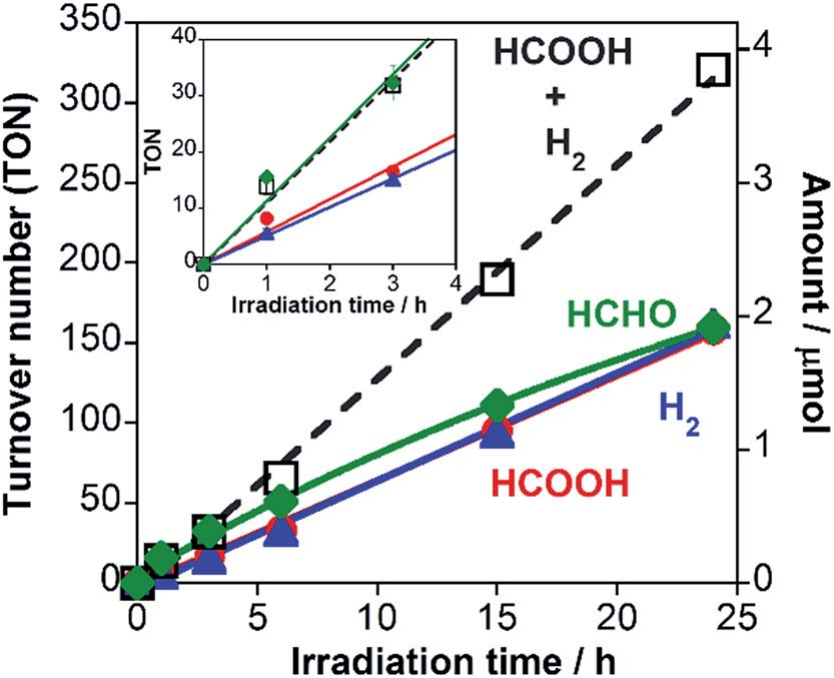
Time courses of HCOOH (red), H_2_ (blue) and HCHO (green) formation along with the sum of HCOOH and H_2_ (black broken line) in the photocatalytic reaction: RuRu/Ag/TaON (4 mg) in a H_2_O–MeOH (4 : 1 v/v) mixed solution (4 mL) was irradiated by visible light (*λ* > 400 nm) under a CO_2_ atmosphere. Inset shows enlarged time courses until 4 h irradiation.

We have already reported that RuRu/Ag/TaON can use methanol as a reductant for CO_2_ reduction in pure methanol.^14^ This is important because CO_2_ reduction with methanol oxidation producing HCOOH as a reduced product of CO_2_ and HCHO as an oxidized product of methanol (eqn (2)) is an endergonic reaction (Δ*G*^0^ = +83 kJ mol^−1^); in other words, the visible-light energy is converted into chemical energy *via* the photocatalytic CO_2_ reduction reaction. As the next step, in this study, we investigated whether the same endergonic CO_2_ conversion reaction can proceed even in aqueous solution. Fig. 6 shows a time course of the TONs of both reduction products (HCOOH and H_2_) and an oxidation product (formaldehyde) in a photocatalytic reaction using RuRu/Ag/TaON in a H_2_O–MeOH mixed solution (4 : 1 v/v) without any other reductants. HCOOH and H_2_ were produced continuously and TON_HCOOH_ reached 17 at 3 h of irradiation. Formaldehyde was also formed, whose produced amount corresponded to the total of HCOOH and H_2_ (Fig. 6 inset). This indicates that the overall reaction of the CO_2_ reduction can be represented in eqn (2).

2 CO_2_ + CH_3_OH → HCOOH + HCHO

**Fig. 7:**
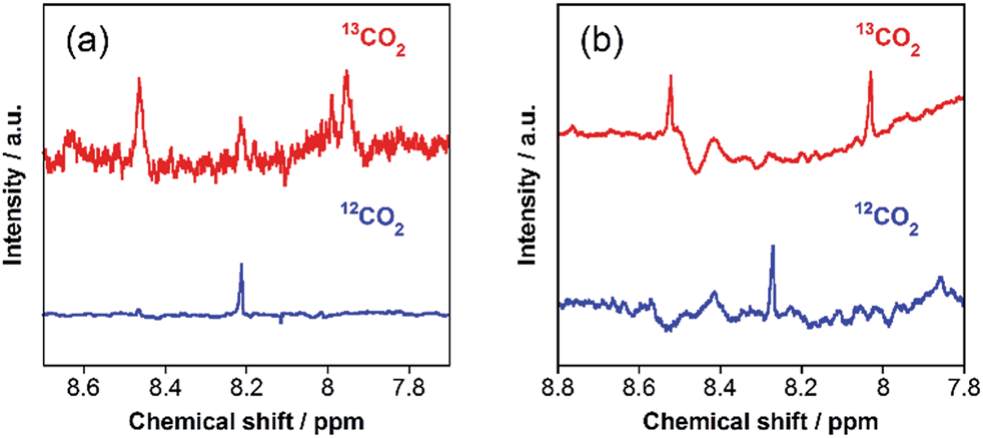
^1^H NMR spectra of reaction solutions (2 mL) containing RuRu/Ag/TaON (8 mg) in (a) H_2_O–MeOH (4 : 1 v/v) and (b) H_2_O–iPrOH (4 : 1 v/v) after a 48 h irradiation with visible light (*λ* > 400 nm) under ^13^CO_2_ (red) and ^12^CO_2_ (blue) atmospheres.

However, further irradiation induced less production of formaldehyde than the sum of HCOOH and H_2_ (Fig. 6). We employed a ^13^CO_2_ labelling experiment to clarify the carbon sources of HCOOH. Fig. 7a shows the ^1^H NMR spectrum of the filtered reaction solution after irradiation for 48 h; a doublet signal with ^1^*J*_CH_ = 204 Hz and a singlet at 8.21 ppm are attributed to the methine protons of H^13^COOH and H^12^COOH, respectively. From this spectrum, we estimated that the main carbon source of HCOOH was CO_2_ (86%), although there were other carbon sources (14%). To gather information on the other carbon sources, a similar photocatalytic reaction was conducted using 2-propanol (i-PrOH) instead of methanol. This photocatalytic system also yielded HCOOH with TON_HCOOH_ = 58 after 15 h of irradiation but did not give any HCHO. Fig. 7b shows the result of a ^13^CO_2_ labelling experiment using i-PrOH as the reductant; the ^1^H NMR spectrum of the filtered reaction solution after 48 h irradiation exhibits that the HCOOH was completely produced from CO_2_. Therefore, when methanol was used as the reductant, partial HCOOH produced in the photocatalytic reduction was probably generated by further oxidation of HCHO, which was produced by oxidation of the methanol. This is also supported by the following result: the photocatalytic oxidation of MeOH using TaON as a photocatalyst and AgNO_3_ as a sacrificial oxidant in aqueous solution containing MeOH yielded not only HCHO as a main product but also HCOOH as a minor one (Fig. S8, ESI†). This minor formation process of HCOOH should contribute to determining the product distribution after a certain amount of HCHO was generated in the reaction solution. As described above, the ‘mismatch’ between the amount of HCHO and the total amount of HCOOH and H_2_ was initially observed after a 6 h irradiation, and a longer irradiation increased this mismatch.

## Experiments

### General procedures

UV-vis absorption spectra were measured with a JASCO V-565 spectrophotometer. X-ray diffraction was measured with a Rigaku MiniFlex 600. FT-IR spectra were measured at 1 cm^−1^ resolution with a JASCO FT/IR-610 spectrophotometer. Emission spectra were measured at 298 ± 0.1 K with a JASCO FP-6500 spectrofluorometer. Emission lifetimes were measured with a Horiba FluoroCube 1000U-S time-correlated single-photon-counting system (the excitation source was a nano-LED 440L, and the instrument response was less than 1 ns).

### Materials

RuRu/Ag/TaON was synthesized according to a literature procedure.^14^ Briefly, an AgNO_3_ (137 μM) aqueous solution (10 mL) was added dropwise to a dispersion (100 mg) of TaON in water (10 mL), followed by stirring for 2 h. Then the suspension was evaporated and the residue was heated at 473 K for 1 h under a H_2_ atmosphere to obtain 1.5 wt% Ag-modified TaON (Ag/TaON). Then, a moderate amount of Ag/TaON was soaked in an acetonitrile solution of the Ru(ii) binuclear complex (RuRu) for 3 h to obtain RuRu/Ag/TaON. The adsorption amount was estimated by the UV-vis absorbance changes of the solution before and after soaking (Fig. S4, ESI† shows an example of a RuRu adsorbed sample with a loading amount of 3 μmol g^−1^).

Ag/Al_2_O_3_ and Ag/TiO_2_ were prepared by the same impregnation–hydrogenation method followed by adsorption of RuRu as RuRu/Ag/TaON for Al_2_O_3_ (AEROXIDE Alu C, AEROSIL) and TiO_2_ (AEROXIDE TiO_2_ P25, AEROSIL), respectively.

Tap water was purified using a Millipore Elix Essential 3 UV system and used on the same day. Methanol was used after distillation. Absolute 2-propanol was purchased from Kanto Chemical Co., Inc. and used without purification. Other materials were reagent-grade quality and were used without further purification.

### Photocatalytic reactions

A suspension of photocatalyst (4 mg) in a reaction solution (4 mL) was prepared in an 8 mL test tube (i.d. = 8 mm) and purged with CO_2_. The suspensions were irradiated by stirring using a photocatalytic reactor (Koike Precision Instruments) at *λ* > 400 nm with a high-pressure Hg lamp combined with a NaNO_2_ aqueous solution filter. The temperatures of the solutions were controlled at 298 ± 2 K using an EYELA constant temperature system (CTP-1000) during irradiation. The quantum yield for HCOOH and H_2_ formation was evaluated in a reaction cell containing RuRu/Ag/TaON (30 mg) in a reaction solution (10 mL), which was irradiated with 400 nm monochromatic light using a 300 W Xe-lamp (Asahi Spectrum MAX-303) with a band pass filter (fwhm = 10 nm). The gaseous reaction products, *i.e.* CO and H_2_, were analyzed by a GC-TCD (GL Science GC 323). HCOOH in the liquid phase was analyzed by a capillary electrophoresis system (Otsuka Electronics Co. Capi-3300I). HCHO was quantitated by a colorimetric analysis following a reported procedure.^14^

We evaluated the photocatalytic activity of the hybrids by using turnover number (TON, eqn (3)), selectivity (eqn (4)) and external quantum efficiency (*Φ*, eqn (5)).3
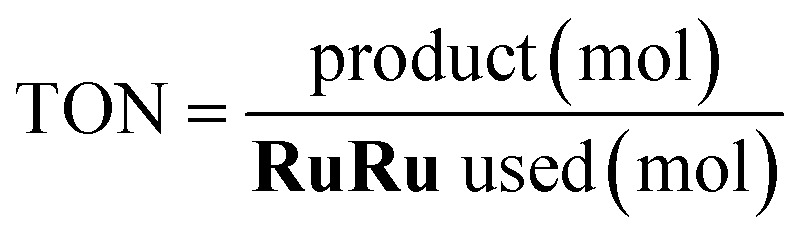
4
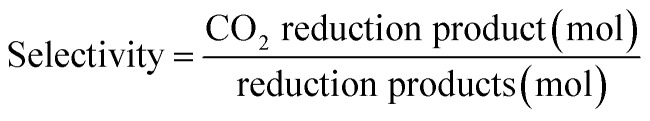
5
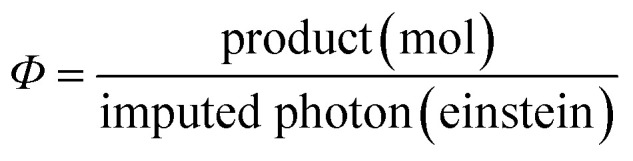


### ^13^CO_2_ labelling experiments

^13^CO_2_ labelling experiments in EDTA·2Na (10 mM) aqueous solution were performed using a dispersion of RuRu/Ag/TaON (4 mg) in aqueous solution (1 mL) containing EDTA·2Na (10 mM) in a reaction cell. The cell was degassed using the freeze–pump–thaw method, and then ^13^CO_2_ (99%, 703 mmHg) was introduced into it. For the photocatalytic system in H_2_O–MeOH mixed solution, a suspension of RuRu/Ag/TaON (8 mg) in a H_2_O–MeOH (2 mL, 4 : 1 v/v) mixed solution in an 8 mL test tube was purged with ^13^CO_2_ (99%) for 20 min. The suspensions were irradiated using a photocatalytic reactor (Koike Precision Instruments) at *λ* > 400 nm with a high-pressure Hg lump combined with a NaNO_2_ aqueous solution filter. After photolysis, the reaction solution was analyzed by ^1^H NMR by using a JEOL ECA400II (400 MHz) system with a No-D technique following filtration.

## Conclusions

A hybrid of a supramolecular photocatalyst with both Ru(ii) photosensitizer and catalyst units, and Ag-loaded TaON photocatalyzed CO_2_ reduction, even in aqueous solution; step-by-step photoexcitation of the Ru(ii) photosensitizer unit and TaON could induce both strong reducing and oxidizing power in the hybrid photocatalyst, and relatively efficient CO_2_ reduction giving HCOOH proceeded with high durability in aqueous solution containing EDTA·2Na as an electron donor. This Z-scheme-type hybrid photocatalyst could also induce reduction of CO_2_ with methanol as the reductant giving HCOOH and HCHO even in aqueous solution, where the visible-light energy was converted into chemical energy (Δ*G*^0^ = +83 kJ mol^−1^).

## Supplementary Material

SC-007-C6SC00586A-s001
