# Supplementary material for: Visible-light-driven CO2 reduction on a hybrid photocatalyst consisting of a Ru(ii) binuclear complex and a Ag-loaded TaON in aqueous solutions
Source: Chem Sci. 2016 Mar 23;7(7):4364–71. doi: 10.1039/c6sc00586a (PMC6014105; doi:10.1039/c6sc00586a)
Supplement: SC-007-C6SC00586A-s001 [file SC-007-C6SC00586A-s001.pdf]

Electronic Supplementary Material (ESI) for Chemical Science.
This journal is © The Royal Society of Chemistry 2016

Visible-Light-Driven CO_2 Reduction on a Hybrid Photocatalyst Consisting of a Ru(II) Binuclear Complex and a Ag-Loaded TaON in Aqueous Solutions

*Akinobu Nakada, Takuya Nakashima, Keita Sekizawa, Kazuhiko Maeda and Osamu Ishitani**

Department of Chemistry, Graduate School of Science and Engineering, Tokyo Institute of Technology, 2-12-1-NE-1 O-okayama, Meguro-ku, Tokyo 152-8550, Japan.

Supporting Information

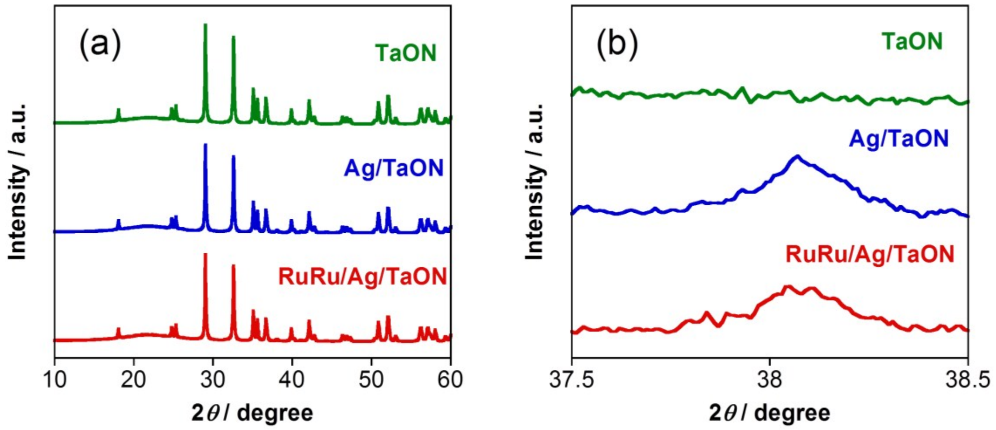

Fig. S1 (a) XRD patterns of **RuRu**/Ag/TaON (red), Ag/TaON (blue), and TaON (green). (b) Enlarged patterns of (a).

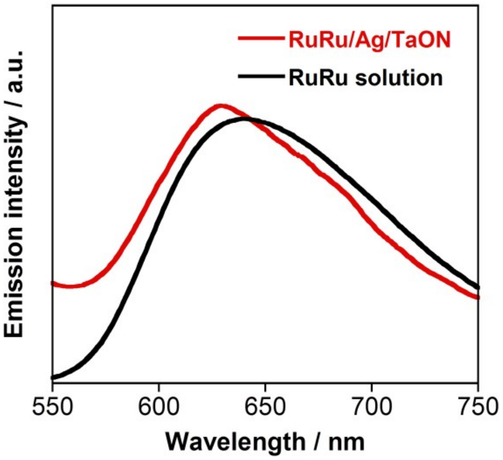

Fig. S2 Emission spectra of **RuRu**/Ag/TaON and **RuRu** in water.

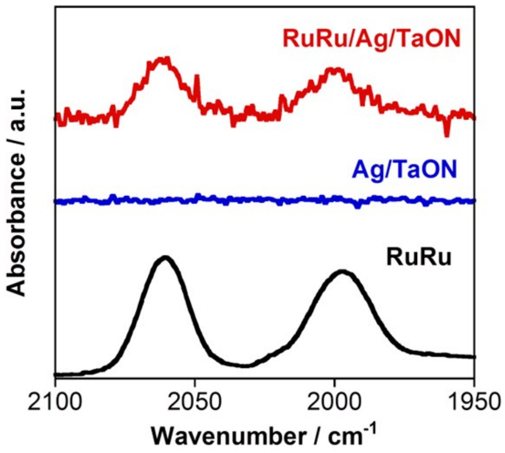

Fig. S3 FT-IR spectra of **RuRu**/Ag/TaON (red), Ag/TaON (blue), and **RuRu** (black) in KBr pallets.

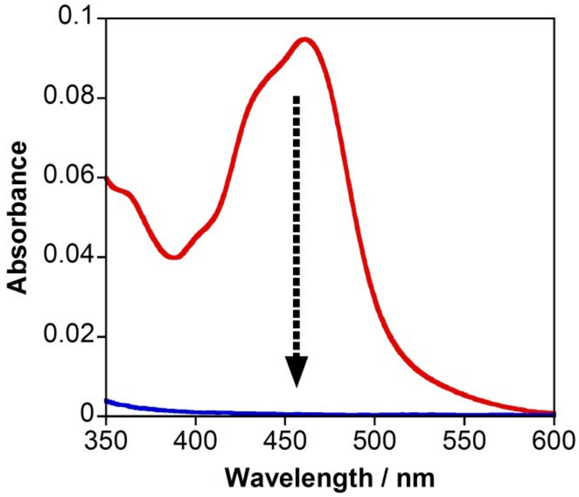

Fig. S4 UV-vis absorption spectra of **RuRu** solution in acetonitrile before (red) and after (blue) soaking with Ag/TaON.

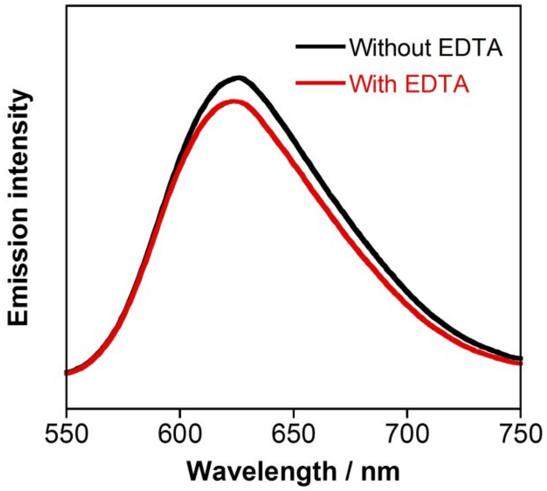

Fig. S5 Emission spectra of **RuRu** adsorbed on Al_2O_3 (4 mg) in aqueous solution (4 mL) without (black) and with (red) EDTA·2Na (10 mM). The excited wavelength was 444.

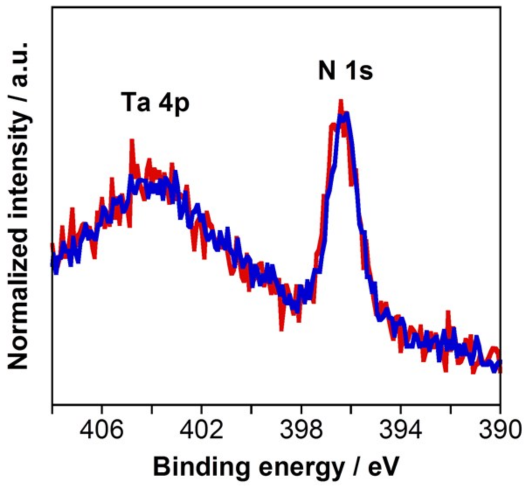

Fig. S6 XPS spectra for Ta 4p and N 1s of **RuRu**/Ag/TaON normalized by the peak area for Ta 4f before (red) and after (blue) photocatalytic reaction corresponding to Table 1, entry 1.

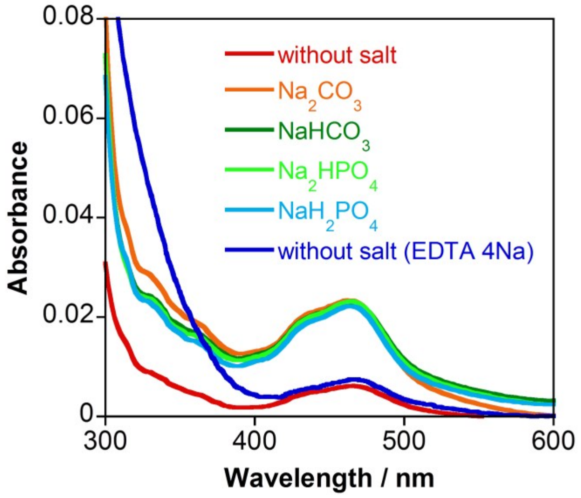

Fig. S7 UV-vis absorption spectra after photocatalytic reaction corresponding to Table 2 entry 1-4, 6, and 11.

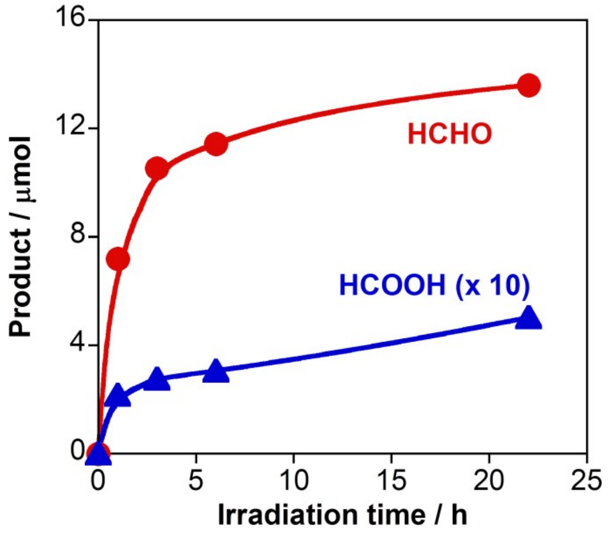

Fig. S8 Time-dependent produced amounts of HCHO (red) and HCOOH (blue) via photocatalytic MeOH oxidation over TaON (4mg) in $AgNO_3$ (10 mM) aqueous solution (4 mL) with a visible-light (λ > 400 nm) irradiation under an Ar atmosphere.
